# Icariin induces cell differentiation and cell cycle arrest in mouse melanoma B16 cells via Erk1/2-p38-JNK-dependent pathway

**DOI:** 10.18632/oncotarget.20118

**Published:** 2017-08-10

**Authors:** Dan Wang, Wenjuan Xu, Xiaoyu Chen, Jichun Han, Lina Yu, Caixia Gao, Wenjin Hao, Xiaona Liu, Qiusheng Zheng, Defang Li

**Affiliations:** ^1^ School of Integrated Traditional Chinese and Western Medicine, Binzhou Medical University, Yantai 264003, Shandong, China

**Keywords:** B16 cells, cell-cycle arrest, differentiation, icariin, Erk1/2-p38-JNK-dependent pathway

## Abstract

Icariin (ICA) is a major component isolated from Epimedium brevicornum. Emerging evidence shows that ICA can inhibit tumor cell proliferation, invasion and migration. However, the anti-cancer effect of ICA on B16 cells has not been fully investigated. Here we found that the proliferation of B16 cells was inhibited by ICA in a concentration- and time-dependent manner, and the colony formation of B16 cells was also inhibited by ICA in a concentration-dependent manner. Further study showed that the melanin content was increased and the tyrosinase (Tyr) activity was enhanced after ICA treatment in B16 cells. Furthermore, compared with the control group, the mRNA levels of Tyr, Trp1 and Trp2 and the protein level of MITF were increased in ICA-treated B16 cells. In addition, the percentage of G0/G1 phase cells was increased and the protein levels of Cyclin A, CDK2 and p21 were decreased in ICA-treated B16 cells. Finally, we found that ICA increased down-regulated the Erk1/2, p-Erk1/2, p38, p-p38, and p-JNK protein levels in B16 cells when compared with the control group. Taken together, these results indicated that ICA could induce B16 cell differentiation and cell cycle arrest at G0/G1 phase through inhibiting Erk1/2-p38-JNK-dependent signaling molecules.

## INTRODUCTION

Melanoma, as the most serious skin cancer, is often considered one of the most aggressive human cancers [1]. Moreover, melanoma is the most common malignant tumor in skin tumors, which is prone to distant metastasis [2]. Although the survival rate of melanoma patients has been greatly improved and its incidence is rapidly increasing throughout the world, especially in the United States, the number of people newly diagnosed with melanoma has increased over the last 10 years [3]. Melanoma development is multifactorial, and its occurrence is related to several different factors such as sun exposure, fair pigmentation, family history, and so on [4]. Melanoma cells can survive in extreme environmental conditions and it is characterized by genomic instability, moreover, cutaneous melanomas are often difficult to treat when diagnosed in advanced stages [5]. Although primary melanoma has a high cure rate in early diagnosis of surgical resection, the survival rate of patients with visceral metastases is only a few months. Therefore, the discovery of new therapeutic agents and melanoma drug targets has become the urgent matter.

Tumor differentiation therapy is a new approach to cancer therapy. Its basic feature is not to kill cells but to induce tumor cells to differentiate into normal or near normal cells. That is, under the action of some chemical agents, some tumor cells appear similar to normal cell phenotype, some of the normal cells to restore some of the features. In recent years, several discoveries reported have revealed a significant breakthrough in the melanoma field-breakthroughs that make a breakthrough in the original treatments and with new treatment strategies to produce clinical benefit of great worth [1]. Cutaneous melanomas can express a variety of immunogenic differentiation melanoma-associated antigens (MAAs), and these antigens play an important role in the outcome of melanoma [6]. The advent of molecular and cellular techniques has led to better properties of tumor cells, revealing the presence of heterogeneous subpopulations of melanoma and cell signaling pathways in melanoma have led to the development of new targeted drugs [7]. Recent studies show some genes/proteins, such as CDKN2A, Ink4a/Arf and MAPK pathway molecules, play a role in melanoma differentiation. The results suggest that the p38 MAPK signaling pathway was involved in the melanogenesis of apigenin, that the activation of p38 MAPK and the up-regulation of MITF contribute to the melanogenesis of apigenin in B16 cells [8].

Icariin is a dry leaf extract of *Epimedium sagittatum, Epimedium pubescens, Wushan epimedium* and *Korean Epimedium*. Icariin can increase blood flow, promote hematopoietic function, immune function and bone metabolism and so on. Icariin has a broad spectrum of anticancer effects, such as inhibiting tumor growth [9], inhibiting tumor cells invasion and migration [10], inducing S-phase arrest and apoptosis in medulloblastoma cells [11]. ICA also exert a number of beneficial cellular effects, including promoting apoptosis, osteogenic differentiation and upregulating extracellular matrix synthesis [12]. It has been reported that ICA could significantly stimulate cardiac differentiation of ES cells *in vitro* [13]. Although recent study suggests that ICA can induce B16 melanoma tumor cells apoptosis *in vitro* and inhibit tumor growth and metastasis *in vivo* [14], the effect of ICA on cell differentiation and cell cycle progression has not been reported. In this study, we examined that whether ICA could influence cell differentiation and cell cycle progression in B16 cells. The data indicated that ICA could induce B16 cell differentiation and cell cycle arrest at G0/G1 phase through inhibiting Erk1/2-p38-JNK-dependent pathway.

## RESULTS

### ICA inhibits the proliferation of B16 cells

**Figure 1:**
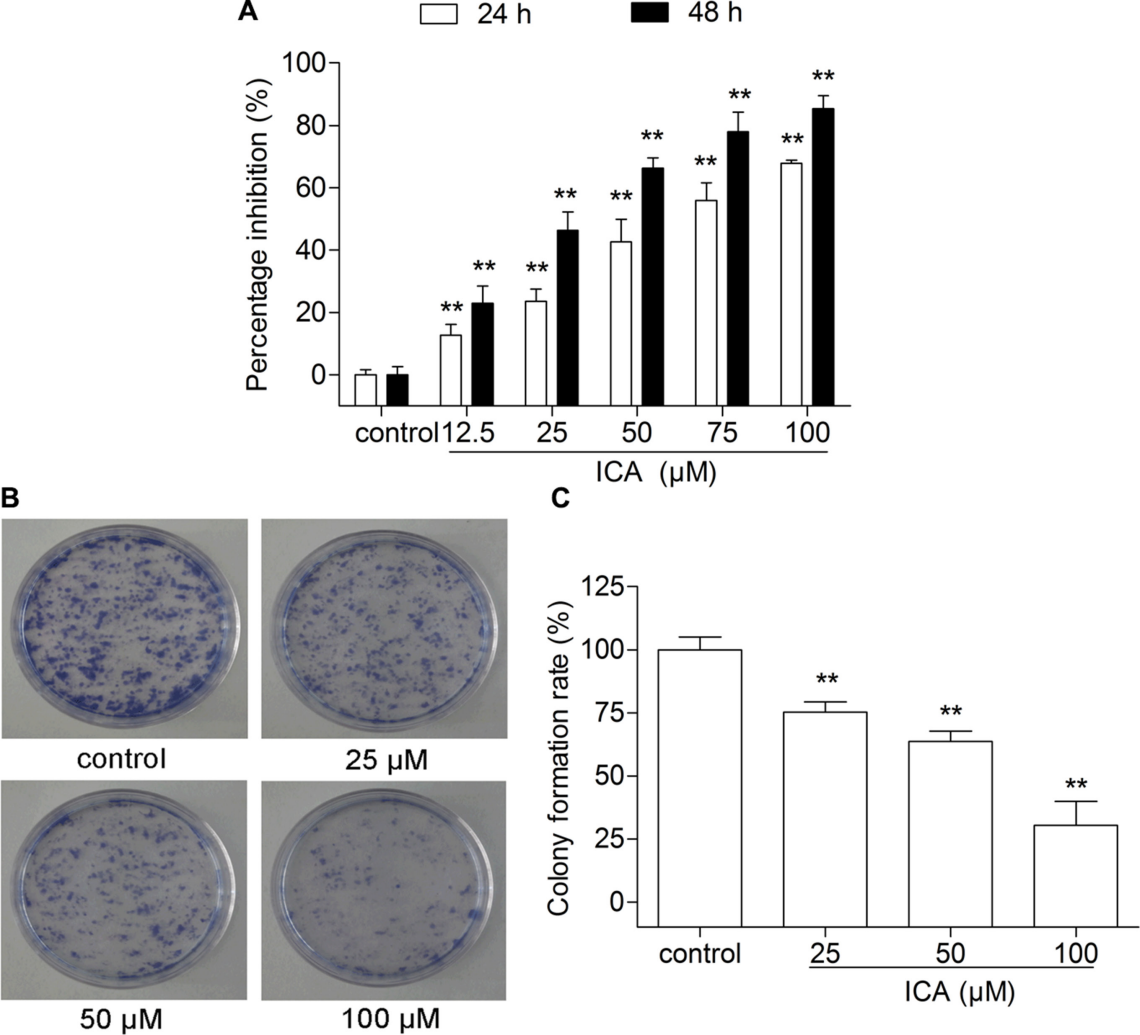
The effect of ICA on B16 cell proliferation and cell colony formation (**A**) The inhibition rate was determined by MTT assay after 24 or 48 h of ICA treatment. (**B**) Representative images of cell colonies after Giemsa staining. (**C**) The values of colony formation inhibition rate among the four groups. All data are presented as the mean ± S.D. of three independent experiments. ^**^P < 0.01 compared with control group.

After treatment with the different concentrations (12.5, 25, 50, 75 and 100 μM) of ICA for 24 or 48 h, B16 cell proliferation was significantly inhibited by ICA in a concentration- and time-dependent manner. Compared with the control group cells, the viability of ICA-treated B16 cells was decreased by 22.93 ± 4.53%, 46.35 ± 4.78%, 66.32 ± 2.64%, 77.97 ± 5.07% and 85.30 ± 3.14%, respectively, at the concentration of 12.5, 25, 50, 75 and 100 μM after 48 h treatment (Figure 1A). Colony formation assay is an *in vitro* cell survival assay based on the ability of a single cell to proliferate into a colony [15]. ICA also inhibited B16 cell colony formation in a concentration-dependent manner (Figure 1B–1C).

### ICA induces melanogenesis through increasing MITF protein expression in B16 Cells

**Figure 2:**
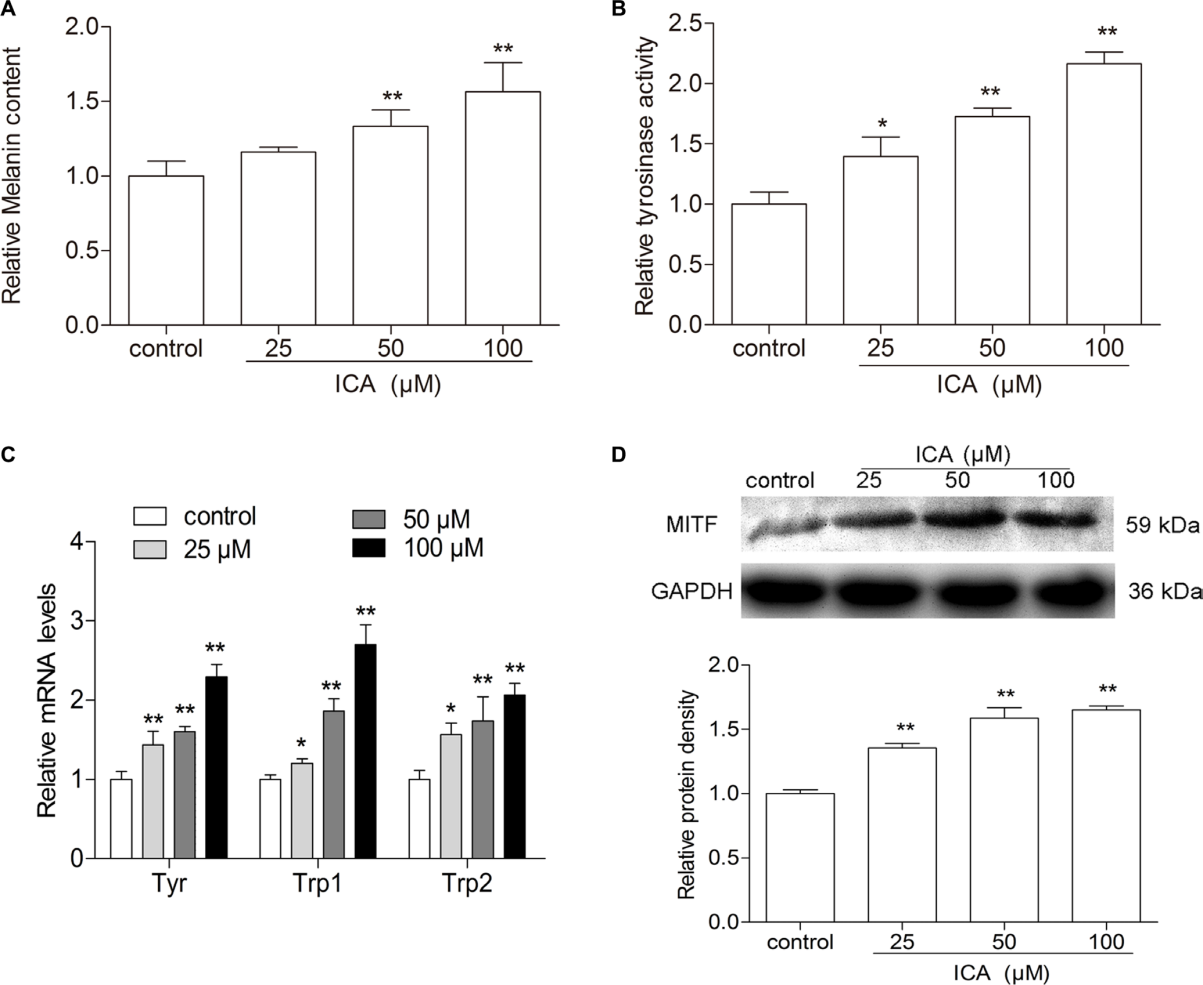
The effect of ICA on melanin content and tyrosinase activity in B16 cells (**A**) The cells were incubated with different concentrations (25, 50, and 100 μM) of ICA for 24 h, melanin contents in B16 cells were measured by colorimetric assay. (**B**) Tyrosinase activity was measured in colorimetric method. (**C**) Quantitative analysis of the mRNA levels of Tyr, Trp-1, Trp-2 by RT-qPCR. (**D**) The protein level of MITF was examined by Western blot. All data are presented as the mean ± S.D. of three independent experiments. ^*^P < 0.05, ^**^P < 0.01 compared with control group.

As we know, melanogenesis is a principal parameter of differentiation in melanoma cells. To confirm that whether ICA could induce B16 cell differentiation, the melanin content was determined in B16 cells by the classical colorimetric method. After 24 h treatment, the levels of melanin were remarkably increased in all ICA-treated group when compared with control group (Figure 2A). Meanwhile, the activity of tyrosinase, a key enzyme in melanin synthesis [16], is significantly increased in B16 cells after different concentrations of ICA (Figure 2B). Moreover, the melanin content is one of symbol of B16 cell differentiation and the melanogenic enzymes, e.g. tyrosinase (Tyr), tyrosinase-related protein 1 (Trp-1) and tyrosinase-related protein 2 (Trp-2) are thought to be the major enzymes in melanin biosynthesis, we further examined the expression levels of melanogenic enzymes including Tyr, Trp-1, and Trp-2 in B16 cells after exposed to ICA. Real time analyses showed that ICA could increased the expression of Tyr, Trp1, Trp2 (Figure 2C). Owing to MITF is a master regulator of melanocyte development, function and survival and it can transcriptionally regulate the tyrosinase family genes TYR, TRP-1, TRP-2 [17, 18], so we also examined the protein expression of MITF and found that ICA could significantly increased the MITF protein expression (Figure 2D).

### ICA induces G0/G1 phase arrest in B16 cells

**Figure 3:**
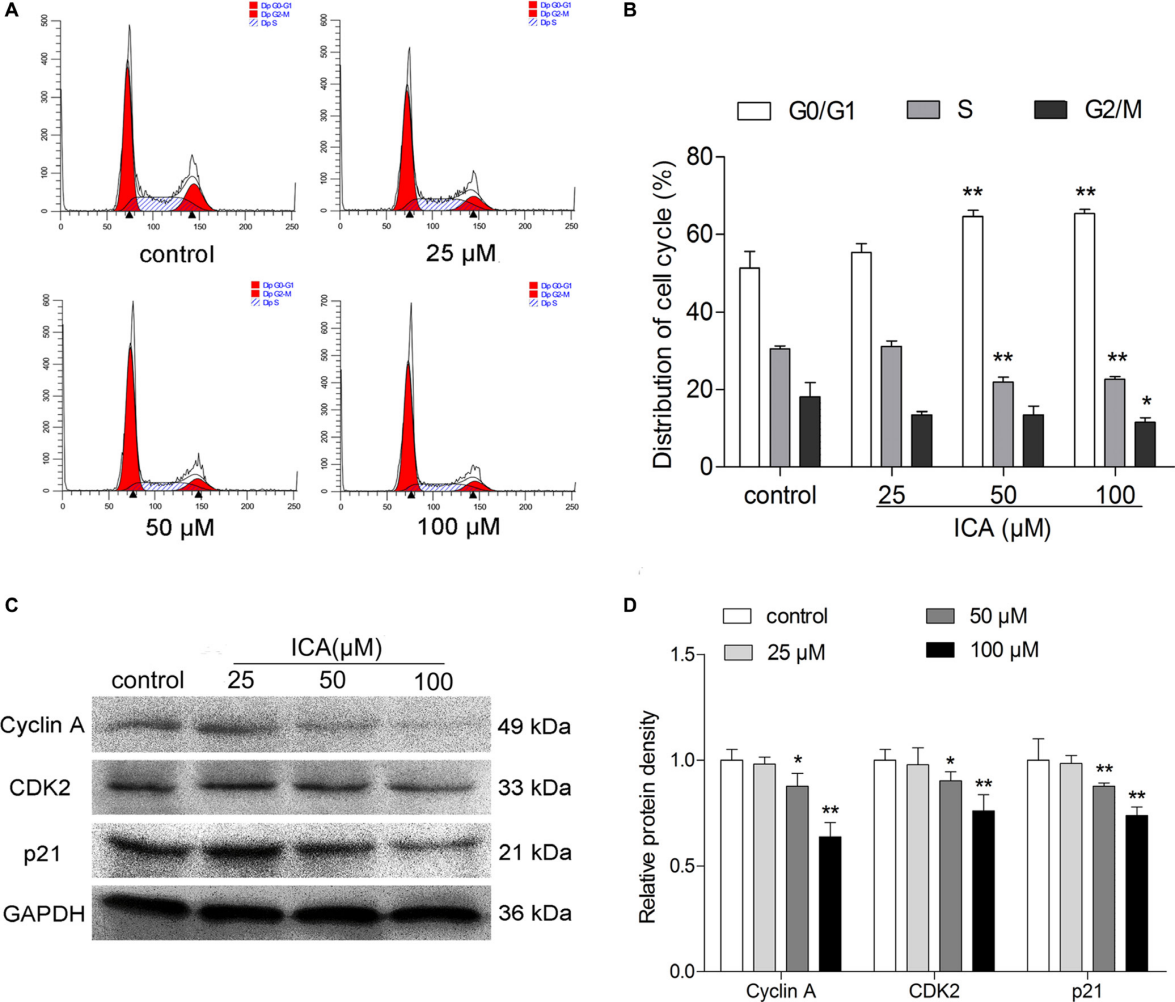
The effect of ICA on cell cycle distribution of B16 cells B16 cells were incubated with 25, 50 and 100 μM of ICA for 24 h. (**A**) Cells were harvested to measure the cell cycle distribution by flow cytometry. (**B**) Quantitative analysis of cell cycle distribution after ICA treatment. (**C**) The protein level of Cyclin A, CDK2 and p21 were examined by Western blot. (**D**) Quantitative analysis of the protein level of Cyclin A, CDK2 and p21 in ICA-treated B16 cells. All data are the mean ± S.D. of three independent experiments. ^*^P < 0.05, ^**^P < 0.01 compared with control group.

Furthermore, the cell cycle distribution of ICA-treated B16 cells was measured by flow cytometer after PI staining. The data showed that the percentage of B16 cells at G0/G1 phase was significantly higher in ICA-treated (50 and 100 μM) cells than that in control group cells (Figure 3A). Especially, after 24 h treatment, ICA (100 μM) caused an remarkably increase at G0/G1 phase (65.44 ± 0.93%) compared with the control group (51.34 ± 3.48%), a decrease at G2/M phase (11.56 ± 0.94%) compared with the control group (18.14 ± 2.94%) and S phase (23.00 ± 0.05%) compared with the control group (30.52 ± 0.57%) (Figure 3B). Based on the above result, we further detected the protein expression of cell cycle-related molecules. Compared with the control group, the expression of CDK2, Cyclin A and p21 were remarkably decreased in ICA-treated B16 cells (Figure 3C–3D).

### ICA decreases the expression levels of Erk1/2-p38-JNK-dependent signaling molecules in B16 cells

**Figure 4:**
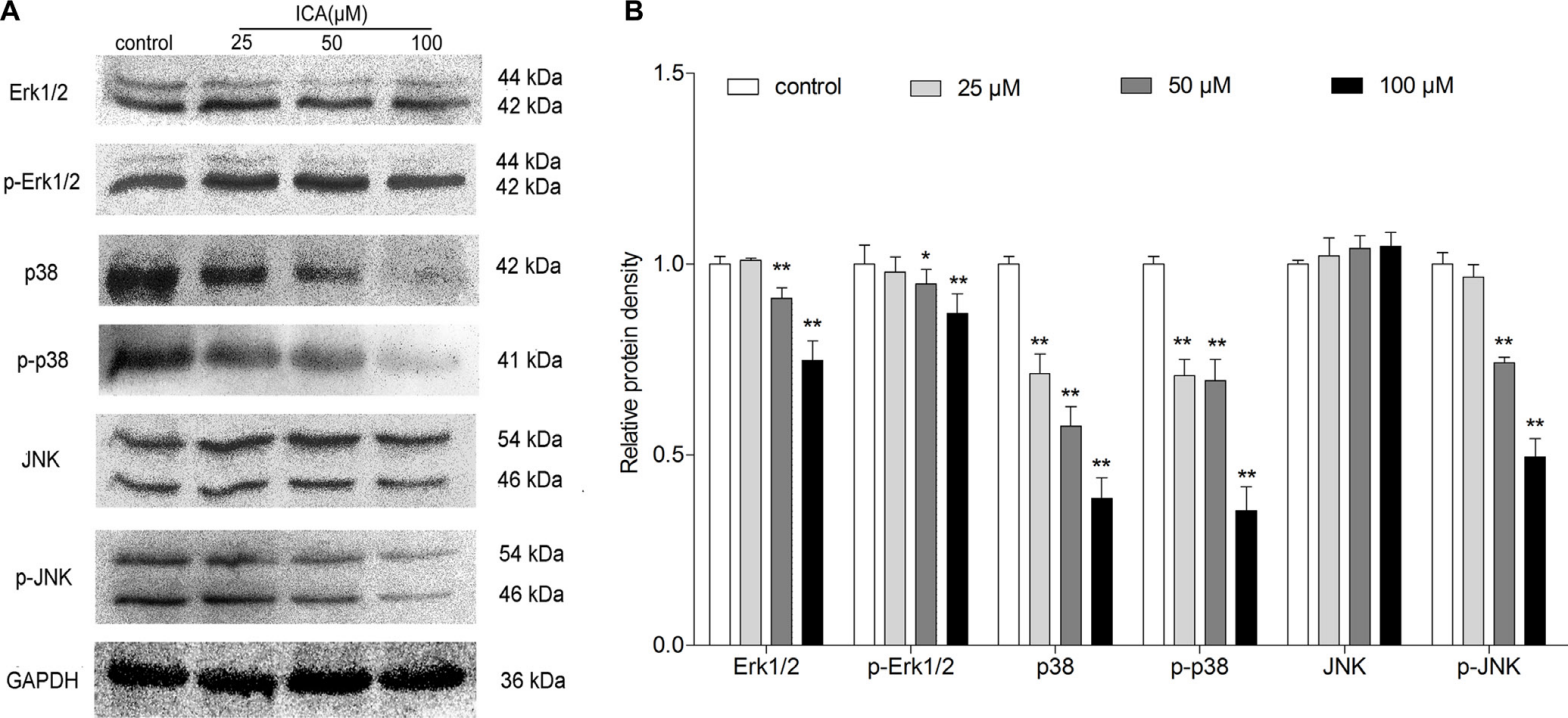
The effect of ICA on protein expression levels of MAPK signaling molecules in B16 cells Cells were exposed to ICA at 25, 50 and 100 μM for 24 h. (**A**) The protein level of Erk1/2, p-Erk1/2, p38, p-p38, JNK and p-JNK were examined by Western blot. (**B**) Western blot analysis of Erk1/2, p-Erk1/2, p38, p-p38, JNK and p-JNK protein level in ICA-treated B16 cells. All data are the mean ± S.D. of three independent experiments. ^*^P < 0.05, ^**^P < 0.01 compared with control group.

To determine whether Mitogen Activated Protein Kinases (MAPK) pathway was involved in ICA-induced cell differentiation and cell cycle arrest of B16 cells, western blot assay was performed to investigate the change of the protein expression of MAPK signaling molecules, e.g. extracellular signal regulated kinase (ERK), c-Jun amino-terminal kinases (JNK) and p38 MAPK kinase. Western blot analysis demonstrated that ICA could significantly decreased the Erk1/2, p-Erk1/2, p38, p-p38, and p-JNK protein level in B16 cells, but show no significant effect on the level of JNK (Figure 4A–4B).

## DISCUSSION

As we all know, malignant melanoma is a tumor that respond poorly to chemotherapy and melanoma is the most aggressive form of skin cancer [15]. Moreover, malignant melanoma is a high malignancy, metastasis and high mortality of skin cancer, there is a growing trend in recent years, although chemotherapy achieved a certain effect, they have limitations and high toxicity. So, it is urgent to find a novel and less toxic potentially candidate drugs and a new treatment for melanoma therapy. ICA has a broad spectrum of established pharmacological functions, including antioxidant effect and induction of cardiomyocyte differentiation and it also shows an anti-inflammatory effect on LPS-treated murine chondrocytes [19]. In this study, we found that ICA could inhibit cell proliferation and reduced colony formation in a concentration- and time-dependent manner. This is important because uninhibited migration of melanoma leads to aggressive phenotype and high mortality rates of patients [20]. So, we carried out further experiments to explore the mechanism of ICA on B16 cell proliferation.

Melanin content and tyrosinase activity are primary molecular markers of melanoma cells differentiation. In addition, the formation of melanin is an important manifestation of the differentiation of melanoma cells [21]. In this study, melanin content and the tyrosinase activity were significantly increased in ICA-treated cells compared with that of control cells. These results showed that ICA induced a differentiation program in B16 cells. We further examined the expression levels of melanogenesis-related proteins including Tyr, Trp-1, and Trp-2 in B16 cells after exposed to ICA. The data showed that ICA could increased the expression of Tyr, Trp1, Trp2. This was another strong evidence demonstrated that ICA could induce B16 cell differentiation. Owing to MITF is critical in transcriptional activation of genes required for tyrosinase, Tyr, Yrp1, and Trp2 and the differentiation of melanocytes [22]. And it has been previously reported that MITF is a master gene regulating differentiation of melanocytes [23]. We found that the protein expression of MITF and found that ICA could increased the MITF protein level, which is consistent with previous reports. According to the above experiment, we inferred that ICA could increase the melanin content, enhance the tyrosinase (Tyr) activity and increase the mRNA expression of Tyr, Trp1, Trp2 by increasing the MITF protein level. Therefore, it is reasonable to believe that ICA could induce B16 cell differentiation.

The cell cycle engine is a promising target for cancer diagnosis and treatment and its deregulation is the center of abnormal cell proliferation and is characteristic of all cancers [24]. Some studies demonstrated that ICA induces cell cycle arrest at S phase in A549 cells, and down-regulated the expression levels of S regulatory proteins such as Cyclin A and CDK2 [25]. In this study, we found that ICA also inhibited proliferation through inducing a G0/G1 phase arrest in B16 cells. As was previously reported that cell cycle arrest is often linked to cell differentiation [26], is consistent with our results. In further experiments, we found that ICA could decrease the protein levels of Cyclin A, CDK2 and p21 protein level. This is even more proof that ICA can induce a G0/G1 phase arrest.

It is known that MAPK signaling molecules have been identified as critical factors in the development of melanoma, making this cascade an important therapeutic target [27]. Dysregulation of the MAPK pathway significantly affects melanoma and melanoma biology [28]. To further investigate the mechanism of ICA in B16 cells, we examined the protein changes of MAPK signaling molecules. Among them, Erk1/2 signaling pathway is one of the most studied pathways and it is generally believed that Erk1/2 signaling pathway is closely related to melanogenesis [29]. In this study, we found ICA could decrease the protein levels of Erk1/2 and p-Erk1/2. The p38 MAPK is another important for cell differentiation, ectopic activation of p38 MAPK is sufficient to induce myelin breakdown and drives differentiated Schwann cells to acquire phenotypic features of immature Schwann cells [30, 31]. In the experiment, we found that ICA could decrease the protein levels of p38 and p-p38. JNK, also known as the stress activated protein kinase, is activated by three levels of enzymatic cascade reactions and subsequently down-regulating melanogenesis [32]. We found ICA could decrease the protein levels of p-JNK in B16 cells. Through comprehensive consideration to the above results, our findings indicated that ICA could induce the differentiation of B16 cells via Erk1/2-p38-JNK-dependent signaling pathway.

On the other hand, ERK activity needs to be keep increasing during the G1 phase of the cell cycle to proceed to S-phase entry [33]. And the synergistic action of PI3K/Akt signaling and ERK signaling can regulate growth factor-stimulated cell cycle progression [34]. A recent study assumed that p38α nuclear translocation may be involved in inducing G2/M phase arrest and to promoting DNA repair [35]. Unrestrained phosphorylation by JNK can results in abnormal cell cycle progression [36]. In this study, we found that ICA induced G0/G1 phase arrest and decreased the expression of cell cycle-related molecules (Cyclin A, CDK2 and p21). Meanwhile, ICA decreased the protein levels of Erk1/2, p-Erk1/2, p38, p-p38 and p-JNK in B16 cells when compared with the control group. Therefore, these results revealed that ICA could also induce G0/G1 phase arrest in B16 cells via Erk1/2-p38-JNK-dependent signaling pathway.

In summary, it was firstly reported in the present study that ICA, as an anti-tumor drug, induce cell differentiation and cell cycle arrest of mouse melanoma B16 cells via MAPK signal pathway. Thus, ICA may have a potential in the future as an adjuvant therapeutic agent for the treatment of melanoma.

## MATERIALS AND METHODS

### Chemicals and reagents

ICA (molecular weight of 676.65, chemical formula C_33_H_40_O_15_, purity ≥ 98%) was purchased from Chengdu Biopurify Phytochemicals Ltd. (Chengdu, China). ICA was dissolved in DMSO and diluted with fresh medium (DMEM) to achieve the required concentration. The final concentration of DMSO in the fresh medium did not exceed 0.1%, and DMSO has no significant effect on the cell viability at this concentration. DMEM (high glucose) and Fetal bovine serum (FBS) were purchased from Hyclone (Hyclone, UT, USA). DNA Content Quantitation Assay (Cell Cycle) and Penicillin and streptomycin were obtained from Solarbio science & technology co., Ltd. (Beijing, China). Unless indicated otherwise, the other reagents were purchased from Sigma Chemical Company (St. Louis, Missouri, USA).

### Cell culture

Mouse melanoma B16 cells were purchased from Cell Bank of the Committee on Type Culture Collection of the Chinese Academy of Sciences (Shanghai, China). The cells were cultured in DMEM medium supplemented with 10% FBS, 100 U/mL penicillin, and 100 μg/mL streptomycin, in the cell incubator with 5% CO_2_ at 37°C.

### Cell proliferation assay

Effects of ICA on viability of B16 cells were determined using the MTT assay [37]. Firstly, B16 cells were collected after digestion with 0.05% Trypsin-EDTA and cultured in 96-well plates at approximately 8×10^3^ cells per well and incubated for 24 h. Then cells were treated with different concentrations of ICA (25, 50, 75 and 100 μM) for 24 or 48 h. Then, MTT solution (5 mg/mL in PBS, pH = 7.4) was added (10 μL/well) to cultures and placed in the cell incubator with 5% CO_2_ at 37°C for 4 h. Subsequently, 150 μL DMSO was added to each well and plates were put on shaker for 10 min to make the formazan crystal violet dissolved completely, the absorbance of each well was measured at 490 nm by the fluorescence plate reader (Bio-Rad Laboratories, CA, USA). The results reported are means of three independent experiments. Inhibition percentage (IP %) was calculated according to the following formula: Inhibition percentage of cell viability (%) = (1- (OD treated cells/OD control cells)) ×100%.

### Clonogenecity assay

B16 cells were seeded onto 60-mm culture dishes at approximately 200 cells per dish. After allowing the cells to adhere for 24 h, cells were treated with different concentrations of ICA (25, 50 and 100 μM) for 24 h. After a further culture for 14 d, colonies were fixed in methanol and stained with Giemsa staining solution, took pictures with the digital camera. Then the number of clones containing more than 50 cells was calculated under a microscope and the colony formation rate is calculated in accordance with the following formula: Colony formation rate (%) = (colony number / number of inoculated cells) ×100%.

### Determination of melanin content

The cells were placed on 6-well chamber slides at a density of 2×10^5^ cells per slide, and treated with different concentrations of ICA (25, 50 and 100 μM) for 24 h to determine melanin content of B16 cells. After 24 h, removed the culture medium and cells were washed twice with PBS, then B16 cells were collected after digestion with 0.05% Trypsin-EDTA and count the cells. Then 1 × 10^5^ cells per well were dissolved in 0.025 mol/L NaOH (contain 10% DMSO) for 1 h at 80°C. Subsequently transfer the liquid to 96-well plates. The absorbance of each well was measured at 490 nm by the fluorescence plate reader [38]. All the data of the treated groups was normalized by the mean value of the control group.

### Tyrosinase activity assays

Tyrosinase activity was estimated by measuring rate of L-DOPA oxidation, as described previously with slight modification [38, 39]. The cells were placed on 6-well chamber slides at a density of 2 × 10^5^ cells per slide, and treated with different concentrations of ICA (25, 50 and 100 μM) for 24 h to determine tyrosinase activity of B16 cells. After 24 h, the cells were washed with ice-cold PBS twice, collected after digestion with 0.05% Trypsin-EDTA and centrifuged at 800 rpm for 3 min. Then count the cells and 1 × 10^5^ cells per well were washed once with PBS and 100 μL 0.5% sodium deoxycholate were added to each well and incubated at 0°C for 15 min, subsequently mixed with 300 μL of 0.1% L-DOPA in PBS (pH = 6.8) at 37 °C. After 2 h, 100 μL of supernatant were added to each well in 96-well plates, the absorbance at 490 nm was measured. All the data of the treated groups was normalized by the mean value of the control group.

### Cell cycle analysis

Cells at a density of 1.5 × 10^5^ cells/mL were treated with the required ICA concentration of 25, 50 and 100 μM for 24 h. Then the experiment was conducted as previously reported [40]. Cells were collected and fixed in 70% ethanol for 3 h at 4°C. After washing with PBS three times, collected cells and treated with 100 μL RNase A solution, resuspended cells and in water bath for 2 h at 37°C. Then add 400 μL PI staining solution for 3 h at 4°C, protect from light. Results analyzed using the flow cytometer (BD, NJ, USA).

### Quantitative real-time polymerase chain reaction

Cells at a density of 1.5×10^5^ cells/mL were treated with the required ICA concentration of 25, 50 and 100 μM for 24 h. Total RNA was extracted using Trizol reagent (Sangon Biotechnology Co. Ltd., Shanghai, China). 3 μL of total RNA in 20 μL volume was reverse-transcribed to cDNA using ReverAid First Strand cDNA Synthesis Kit. (Thermo Scientific™). The cDNAs, forward primers, reverse primers, and 2×SYBR Green PCRmix constitute the total reaction mixture (20 μL) [41]. The thermocycler parameters were as follows: 95°C for 10 min; 40 cycles of 95°C for 10 s, 57°C for 30 s and 70°C for 20 s. The relative amount of interest genes was calculated according to the following formula: 2^-ΔΔCT^ and ΔΔC_T_ = ΔC_T_(X) -ΔC_T_(Y) (X: the treated sample, Y: the control sample, these two are standardized as endogenous reference values) [41].

### Western blot analysis

Cells in 100-mm culture dishes were treated with the required ICA concentration of 25, 50 and 100 μM for 24 h. Then cells were lysed with RIPA lysis buffer (300 μL per dish). Determined protein concentration and the protein sample was added to 5× sample buffer, then boiled and denatured. 100 V constant pressure electrophoresis to near the bottom of the gel and then electro-transferred onto the nitrocellulose membrane (Amersham Biosciences, New Jersey, USA). The membranes with protein were blocked with 5% skim milk in TBST buffer for 2 h at room temperature, followed by overnight incubation with the primary antibody, respectively, with anti-GAPDH (1:3000), anti-Erk1/2 (1:10000), anti-p-Erk1/2 (1:1000), anti-p38 (1:1000), anti-p-p38 (1:400), anti-JNK (1:1000), anti-p-JNK (1:400), anti-MITF (1:250), anti-Cyclin A (1:400), anti-CDK2 (1:8000), anti-p21 (1:1000), at 4°C overnight. All antibodies were diluted with 5% BSA in TBST buffer. After washing with TBST solution three times, 5 min at a time, incubation with secondary antibodies. Finally, the membranes were incubated with ECL chemiluminescence (Thermo, NY, USA) and UVP chemiluminescence imaging system was used to obtain image information [41, 42].

### Statistical analysis

The data were presented as means ± S.D. from at least three independent experiments and evaluated through the analysis of ANOVA followed by student’s *t*-test. The values of *P* < 0.05 were considered statistically significant. The analyses were performed using SPSS 19.0 statistical software.

## Abbreviations

ICA: Icariin
Tyr: tyrosinase
Trp-1: tyrosinase-related protein 1
Trp-2: tyrosinase-related protein 2
L-DOPA: L-3, 4-dihydroxyphenylalanine
MAPK: Mitogen Activated Protein Kinase
ERK: extracellular signal regulated kinase
JNK: c-Jun amino-terminal kinases
CDK2: Cyclin-dependent kinase 2
MITF: microphthalmia-associated transcription factor
